# The translational potential of m6A RNA methylation in DKD: emerging biomarkers for early detection and targeted therapies

**DOI:** 10.3389/fmed.2026.1886521

**Published:** 2026-07-22

**Authors:** Huihui Liu, Jun Luo, Yinzhong Dai, Chenguang Wu, Yan An, Wei Jiang

**Affiliations:** 1 Second Affiliated Hospital, Heilongjiang University of Chinese Medicine, Harbin, China; 2 Heilongjiang Academy of Chinese Medicine Sciences, Harbin, China; 3 First Affiliated Hospital of Heilongjiang University of Chinese Medicine, Harbin, China

**Keywords:** biomarkers, DKD, early detection, M6A RNA methylation, podocyte injury, translational medicine

## Abstract

Diabetic Kidney Disease (DKD) is a primary cause of end-stage renal disease, characterized by podocyte dysfunction. While current clinical biomarkers including albuminuria and estimated glomerular filtration rate are foundational for diagnosis, they often function as lagging indicators and fail to detect early renal injury. Recently, N6-methyladenosine (m6A) RNA methylation has emerged as a key epitranscriptomic link between hyperglycemia and podocyte injury, offering a novel frontier for diagnostic biomarker discovery. This review shifts the traditional focus from basic molecular mechanisms to the preclinical translational potential of m6A regulators and their downstream targets as early detection tools. Current experimental evidence in cell and animal models indicates that the abnormal upregulation of *METTL3*, *METTL14*, and *FTO* may reshape podocyte fate through complex regulatory pathways. These alterations appear to enhance the stability of harmful transcripts while degrading protective ones. Furthermore, specific regulators and their associated hub genes, such as *CTSS* and *CD53*, show emerging promise as candidate non-invasive biomarkers that correlate with immune infiltration and early renal function decline in preclinical settings, though robust clinical validation remains necessary.

## 1 Introduction

As one of the most prevalent microcirculatory complications, Diabetic kidney disease (DKD) also contributes largely to Chronic Kidney Disease (CKD) and end-stage renal disease (ESRD) (1–3), which brings high costs in both global public health and medical field, it consumes a lot of medical resources (4). About 25%–40% of all diabetic people will develop DKD (5). DKD has recently been recognized as being at the heart of the newly described Cardiovascular Kidney Metabolic (CKM) syndrome. This concept points out that there is a close connection between metabolic problems (obesity and type 2 diabetes) and both cardiovascular and kidney diseases (6). At present, the clinical management of DKD relies heavily on monitoring albuminuria and the estimated glomerular filtration rate (eGFR) (7). However, these traditional metrics possess significant limitations. Albuminuria exhibits high variability and is not universally predictive, as a substantial cohort of patients progress to advanced stages while maintaining normoalbuminuria. Similarly, a decline in the eGFR often manifests late in the disease continuum, serving as a delayed indicator of irreversible renal damage (8). The past decade has witnessed an urgent search for prognostic biomarkers capable of detecting early kidney injury prior to functional collapse.

Despite immense efforts, transitioning discovery phase candidates into regulatory qualified tools remains a formidable challenge. In this context, epitranscriptomics presents an unprecedented opportunity by introducing a dynamic layer of gene regulation that parallels established epigenetic mechanisms such as DNA methylation and histone modification. Foundational studies have established that N6-methyladenosine (m6A) RNA methylation facilitates a rapid, reversible, and post transcriptional response to cellular stress. It is crucial to contextualize how these early discoveries pave the way for understanding specific metabolic alterations in DKD. Within the diabetic environment, it remains a subject of ongoing investigation whether m6A alterations act as direct causal drivers of DKD or serve as secondary adaptive phenomena in response to metabolic stress. Nevertheless, these modifications integrate dynamically with established epigenetic regulators, such as DNA methylation and histone modification, potentially offering synergistic diagnostic value when combined with established DKD biomarker programs. This review aims to systematically evaluate the diagnostic and prognostic value of the m6A regulatory network in DKD, exploring how translating these molecular signatures into clinical practice can reshape early detection and therapeutic strategies.

## 2 The limitations of current clinical biomarkers and the need for epitranscriptomic signatures

The glomerular filtration barrier (GFB) is the main structure in renal filtration, consisting of glomerular endothelial cells, the glomerular basement membrane (GBM), and podocytes (9). In the pathogenesis of DKD, podocyte injury is thought to be the primary cause of proteinuria and disease progression (10). Under diabetic hyperglycemic conditions, podocytes undergo a cascade of pathological changes, including hypertrophy, foot process fusion, epithelial-mesenchymal transition (EMT), apoptosis, and autophagy dysfunction. These interconnected events act synergistically to destroy the integrity of the glomerular filtration barrier and promote glomerulosclerosis (11).

The mechanisms driving podocyte injury are deeply complex, influenced by factors such as advanced glycation end products (AGEs) resulting from hyperglycemia, hemodynamic alterations, oxidative stress, mitochondrial dysfunction, impaired autophagy, inflammation, and ferroptosis (12–14). While treatment approaches centered around RAASi and SGLT2i have successfully slowed DKD progression, these therapies fall short of entirely halting or reversing the course, thus many continue to progress toward end-stage renal disease (15).

Given these complicated and unsatisfying therapeutic outcomes, establishing early diagnostic biomarkers that accurately reflect the initial stages of cellular damage is a critical research priority. M6A stands out as the most abundant and continually alterable modification found within eukaryotic messenger RNAs (16, 17). This process requires three protein types: “writers” catalyze methylation, “erasers” remove methyl groups, and “readers” recognize the mark to carry out functions (18–20). Through m6A, this process affects RNA splicing, translation, transport, and stability, playing a highly precise role in post-transcriptional gene regulation and offering a very sensitive molecular window into early kidney metabolic stress (21).

## 3 m6A regulators as early diagnostic probes in podocyte injury

Epitranscriptomic reprogramming in podocytes represents a pivotal mechanism linking the high glucose environment to cellular dysfunction in DKD. Significant alterations in global m6A levels and the expression profiles of key m6A regulators have been observed in both the glomerular tissue of DKD patients and high glucose (HG)-induced podocyte models (22, 23). A transcriptomic analysis identified as many as 24 differentially expressed m6A-related regulators in the glomerular tissue of DKD patients, indicating that the pathological progression of DKD is accompanied by widespread epitranscriptomic dysregulation (20). Rather than prematurely viewing these alterations as established causal pathogenic mechanisms, we propose that the synergistic multi target regulation by m6A writers and erasers serves as a potential early biological indicator of impending renal failure. It is critical to note that many of the mechanistic pathways discussed herein originate from isolated *in vitro* high glucose models or rodent studies. Consequently, robust clinical validation is strictly required before these pathways can be definitively established as causal paradigms in human DKD.

### 3.1 The pathway involving METTL3 and its downstream targets

In DKD podocytes, current preclinical models suggest that abnormal upregulation of the writers METTL3 and METTL14 is closely associated with cell damage. Experimental studies show that METTL3, the core part of the m6A methylation complex, is consistently elevated in both animal DKD models and podocytes exposed to high glucose (22–25). This upregulation appears to activate various parallel injury pathways via m6A modification, with the specific downstream effects depending on which Reader protein is recruited. First, METTL3 initiates podocyte pyroptosis via the “METTL3-YTHDF1-TRIM29” pathway. Research indicates that METTL3 is upregulated in HG-induced podocytes and significantly increases TRIM29 mRNA levels via m6A modification (24). Mechanistically, the Reader YTHDF1 is recruited to the methylated TRIM29 mRNA, enhancing its stability and thereby upregulating TRIM29 protein expression. This elevation in TRIM29 activates the NLRP3 inflammasome pathway, ultimately leading to podocyte pyroptosis (24). Second, METTL3 promotes apoptosis and dedifferentiation via the “METTL3-IGF2BP2-MDM2” pathway. High expression of METTL3 in DKD models induces aberrant m6A modification of MDM2 mRNA. Unlike YTHDF1, the recruited Reader here is IGF2BP2, which similarly functions by enhancing the stability of MDM2 mRNA. The resulting accumulation of MDM2 protein activates the Notch1 signaling pathway which is a known driver of podocyte injury, thereby inducing apoptosis, inflammation, and a dedifferentiated phenotype (23). Furthermore, METTL3 disrupts mitophagy and exacerbates oxidative stress through the “METTL3-YTHDF2-PINK1” pathway. METTL3-mediated m6A modification also regulates mitochondrial homeostasis (26). Studies confirm that the METTL3/YTHDF2 pathway epigenetically suppresses the expression of PINK1 (24). In this mechanism, the Reader YTHDF2 recognizes m6A-modified PINK1 mRNA and mediates its degradation (27). Since PINK1 is a key initiator of mitophagy (28), its decline impairs this process, leading to the accumulation of dysfunctional mitochondria and excessive ROS generation, which further aggravates oxidative stress and apoptosis (27). Collectively, these findings show that METTL3 upregulation is a potential initiator for podocyte injury in DKD. The specific damage that follows depends on which Reader proteins are involved. In DKD podocytes, the normal balance between stability-promoting IGF2BP proteins and decay-promoting YTHDF proteins is broken. This shift in Reader expression, along with high METTL3 levels, ultimately shapes podocyte fate.

### 3.2 METTL14-Sirt1 pathway regulation of autophagy and inflammation

METTL14, another core part of the m6A methyltransferase complex, also contributes significantly to DKD podocyte injury (29, 30). METTL14, much like METTL3, is upregulated in injured podocytes and then promotes m6A modification of Sirt1 mRNA (31). Sirt1 is a crucial protective NAD-dependent deacetylase (32). METTL14 modifies Sirt1 mRNA with m6A, likely with the assistance of YTHDF2, which leads to its fast breakdown and much less Sirt1 protein (20). The reduction of Sirt1 exacerbates podocyte injury by inhibiting autophagy, promoting apoptosis, and initiating inflammatory responses, ultimately accelerating the development of proteinuria (33–35).

### 3.3 The m6A eraser FTO disrupts the autophagic flux

In DKD podocytes, dysregulation involves not only writers but also the Eraser *FTO* (36, 37). The m6A demethylase *FTO* is selectively overexpressed in human DKD kidney tissue and HG-treated podocytes (38). This represents another key pathway by which podocyte autophagy is inhibited, known as the *FTO*-*ATF3* pathway. High expression of *FTO* causes m6A demethylation of *ATF3* mRNA, resulting in lower methylation levels (39). Paradoxically, this loss of methylation makes the transcript more stable so there is more of the transcription repressor *ATF3* building up. Consequently,* ATF3* systemically silences major autophagy genes (*LC3*, *ULK1*, *ATG5*, and *Beclin1*) to block autophagic flux (39). This is particularly detrimental for terminally differentiated podocytes, which rely on autophagy for proteostasis and organelle renewal (40). M6A dysregulation in DKD, whether through the *METTL3*-*PINK1* pathway repressing mitophagy or the *METTL14*-*Sirt1* and *FTO*-*ATF3* axes suppressing global autophagy (39, 41, 42), disrupts cellular clearance and leads to podocyte failure and cell death.

### 3.4 The widespread influence of m6A regulation in podocyte injury

The m6A network touches on most known podocyte injury pathways in DKD, including ferroptosis, which links to renal fibrosis (43). Specifically, the METTL3/YTHDF3 pathway has been shown to stabilize Transferrin Receptor 1 (TfR1) mRNA, increasing iron uptake and fueling ferroptosis (44). Moreover, m6A modifications have been confirmed to regulate critical signaling pathways driving renal fibrosis and podocyte injury, such as TGF-β and Wnt/β-catenin signaling (45–47), while also influencing oxidative stress responses via the Keap1/Nrf2 pathway (47). Collectively, the widespread m6A dysregulation under high glucose metabolic stress represents a significant potential mediator precipitating the collapse of podocyte homeostasis.

## 4 Decoding the m6A landscape: promising biomarkers for clinical translation

The pivotal driving role of the m6A regulatory network in DKD podocyte injury positions it as more than just a key to explaining pathogenic mechanisms; it also provides highly attractive novel targets for the early diagnosis and therapeutic intervention of DKD (48).

### 4.1 Biomarkers linked to the immune microenvironment: CTSS and CD53

Current clinical metrics for DKD, such as albuminuria and estimated glomerular filtration rate (eGFR), are limited by their lagging nature and poor specificity. Given that aberrant m6A modification is an early driver of DKD pathogenesis, m6A signatures offer a route to earlier, more precise molecular stratification. First, m6A regulators’ own expression profiles also have potential for diagnosis. Bioinformatic analysis using Logistic LASSO created a diagnostic model using three m6A regulators: YTHDC1, METTL3, and ALKBH5, it was able to distinguish between early stage DKD patient (49). Second, m6A modifications are closely correlated with the remodeling of the renal immune microenvironment (50). Through the consensus clustering of m6A regulator expression profiles, researchers can differentiate patients with DKD into distinct molecular subsets (49). Studies demonstrate that these molecular subgroups exhibit significantly different immune cell infiltration patterns. For example, Cluster B contains strong infiltrations of immune cells, including activated B cells, CD4 positive T cells, and dendritic cells. This cluster is also rich in apoptotic genes, proliferative genes, and major signaling pathways such as the TGF beta pathway and the PI3K AKT pathway (49, 50). Analysis linking m6A subtypes to immune infiltration identified hub genes, with CTSS and CD53 showing the strongest association. Both are significantly upregulated in DKD patients and mouse models. A key clinical point is that their expression levels correlate negatively with eGFR, the gold standard of renal function (50). These findings validate CTSS and CD53 as promising candidate markers; they not only bridge m6A epigenetics with the immune microenvironment but also serve as quantifiable molecular probes for monitoring renal function deterioration, as evidenced by their significant correlation with eGFR. While CTSS and CD53 present exciting opportunities as candidate biomarkers, their current translational readiness remains experimental (50). It is crucial to evaluate these emerging candidates against established clinical biomarkers for DKD, such as albuminuria, eGFR, Kidney Injury Molecule 1 (KIM-1), Neutrophil Gelatinase Associated Lipocalin (NGAL), and Tumor Necrosis Factor Receptors 1 and 2 (TNFR1/2) (51). Unlike established markers which are clinically validated indicators of functional decline or acute tubular injury, CTSS and CD53 primarily reflect early immune microenvironment remodeling and epitranscriptomic shifts (50). Currently, large scale quantitative data regarding the sensitivity, specificity, and positive predictive value of m6A related markers are lacking. Establishing these quantitative diagnostic thresholds in prospective human cohorts is a critical next step. A comparison of these emerging and established biomarkers is summarized in Table 1. Furthermore, preclinical studies exemplified by the METTL3 inhibitor STM2457 have provided initial hypotheses that targeting m6A modifications could be an effective therapeutic strategy, but these remain strictly within the realm of translational candidates requiring extensive clinical validation (24).

**TABLE 1:** Comparative analysis of established clinical biomarkers and emerging m6A related biomarkers in DKD.

Biomarker category	Specific markers	Pathophysiological reflection	Clinical advantages	Current limitations
Established functional markers	Albuminuria, eGFR	Glomerular filtration barrier disruption, basement membrane thickening, and overall decline in renal filtration capacity.	Universally accessible, standardized global diagnostic criteria, and highly validated for predicting late stage progression and end stage renal disease risk.	Serve as lagging or delayed indicators; high variability in albuminuria; a significant cohort of patients progress to advanced stages while maintaining normoalbuminuria; poor sensitivity for early cellular stress.
Established structural and tubular injury markers	KIM-1, NGAL, TNFR1/2	Proximal tubular epithelial cell stress, acute or chronic tubular structural injury, and tubulointerstitial inflammation.	High sensitivity for detecting acute structural damage and chronic tubulointerstitial injury; easily measurable in non-invasive liquid biopsies such as urine or plasma samples.	Broadly reflect generic tubular injury, generic cell stress, or systemic inflammatory states rather than specific upstream metabolic reprogramming or early podocyte injury early in DKD.
Emerging epitranscriptomic and effector markers	METTL3, FTO, METTL14, CTSS, CD53, LRPPRC, DDX3Y, EGR1, ACC1, lncRNA TUG1 METTL3, FTO, METTL14, CTSS, CD53, LRPPRC, DDX3Y, EGR1, ACC1, lncRNA TUG1	Early post transcriptional epitranscriptomic reprogramming, global or gene specific m6A dysregulation, renal immune microenvironment remodeling, podocyte injury pathway activation, lipotoxicity, and mitochondrial dysfunction.	Potential to detect upstream molecular pathology and dynamic cellular stress responses prior to functional collapse or irreversible histological damage; reflects specific pathway dysregulation and provides targets for synchronized companion diagnostics and therapeutics.	Currently experimental and clinically unvalidated; profiling methods are complex, expensive, and lack high throughput standardization; heavily reliant on preclinical models and bioinformatics without established quantitative diagnostic thresholds such as sensitivity, specificity, and receiver operating characteristic performance in prospective human cohorts.

### 4.2 Markers of tubular stress and metabolic reprogramming

Furthermore, advancements in multi-omics have expanded the repertoire of downstream m6A effector biomarkers to include markers of tubular injury and metabolic remodeling. For example, multi-omics data found *LRPPRC* as a main m6A regulator within DKD tubular cells and could function as an individual prognostic parameter (52). Meanwhile, the aberrant expression of *DDX3Y*, a classic marker of tubular stress, was confirmed to be regulated by *WTAP* and *METTL14* mediated m6A modification (30, 53), offering a new window for non-invasive monitoring of renal stress. Expanding the epitranscriptomic landscape to the non-coding genome, the m6A-mediated stabilization of lncRNA TUG1 bridges epitranscriptomic regulation with mitochondrial homeostasis, establishing a new non-invasive diagnostic target, and its low expression predicting disease progression (54, 55). Furthermore, in specific mechanisms of podocyte injury, *EGR1* and *ACC1*, which serve as key nodes in inflammatory responses and lipid metabolism respectively, were found to be regulated by *FTO* mediated demethylation, making them potential molecular probes for renal inflammation and lipotoxicity (36, 56). Together with *CTSS* and *CD53*, these novel targets construct a multidimensional molecular diagnostic atlas for DKD, promising to compensate for the limitations of traditional clinical metrics.

### 4.3 Emerging preclinical therapies targeting the m6A pathway

Given the pivotal driving role of m6A regulators (particularly writers and erasers) in DKD podocyte injury, targeting the activity of these enzymes has emerged as a new direction in DKD drug development. Targeting the Writer METTL3 represents one of the most promising strategies (57). As previously discussed, METTL3 serves as a critical upstream regulator of multiple injury pathways, including pyroptosis, apoptosis, and mitochondrial autophagy dysfunction (58, 59). One key preclinical study provided a therapeutic hypothesis for targeting METTL3 through intervention with the specific small molecule METTL3 inhibitor STM2457 in a DKD mouse model. The results show that STM2457 improved the podocyte injury and kidney’ s pathology change (24). Mechanistically, this protective effect was achieved by suppressing the activation of the NLRP3 inflammasome and subsequent pyroptosis (60). The success of STM2457 shows that blocking an upstream Writer like METTL3 works well to inhibit multiple downstream pathways. Targeting the Eraser FTO also holds promise. The FTO-ATF3 pathway is a key driver of autophagy impairment in podocytes. Using *in vitro* high glucose models and FTO podocyte-specific knockout (iPFKO) mice, studies show that inhibiting FTO or its downstream effector ATF3 (through genetic or pharmacological approaches) restores podocyte autophagy flux and maintains podocyte structure and function (39). This highlights the therapeutic potential of targeting the FTO-ATF3 pathway to restore podocyte homeostasis and mitigate DKD progression.

## 5 Discussion

**FIGURE 1:**
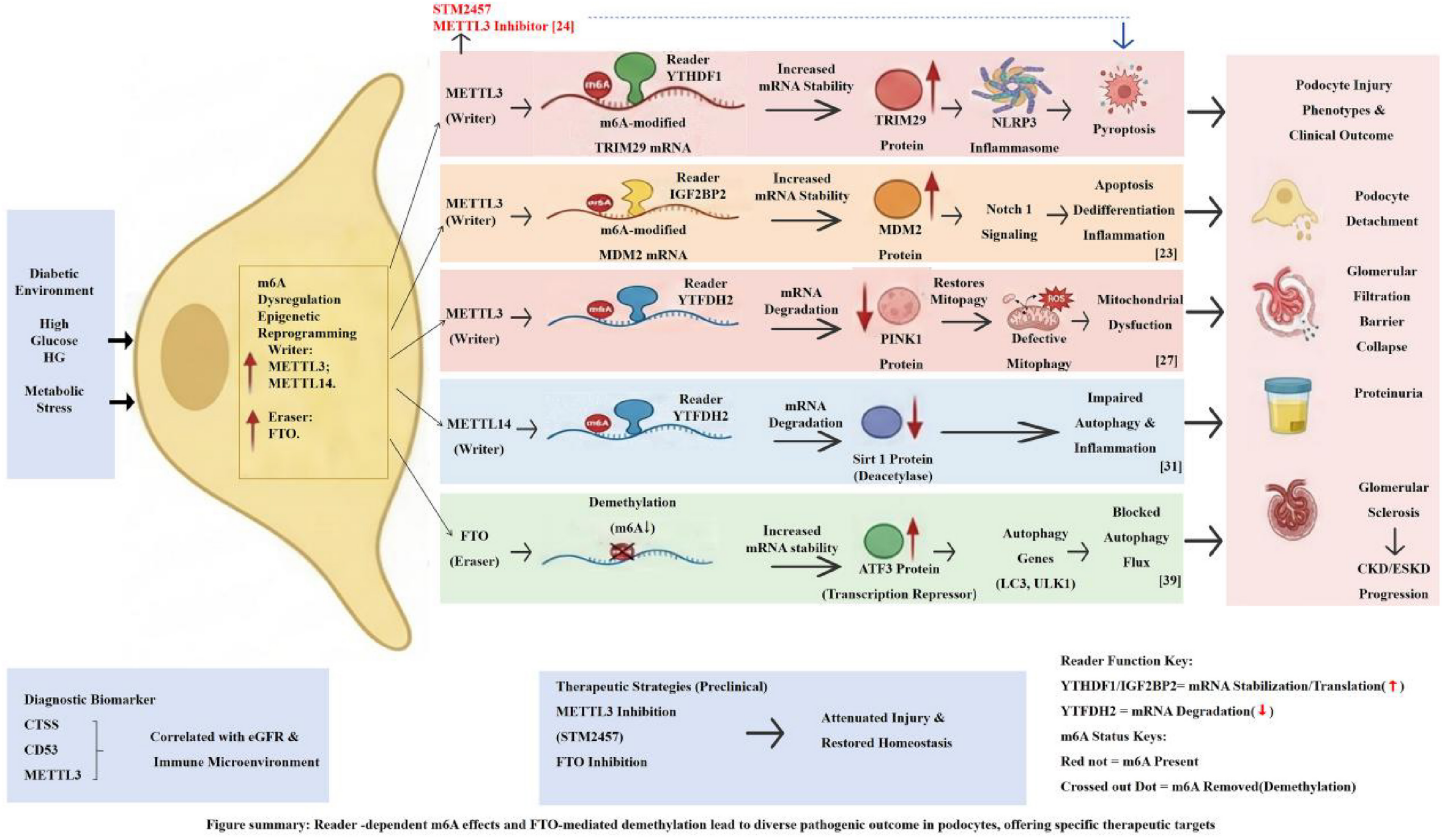
Mechanisms of m6A RNA methylation in diabetic kidney disease podocyte injury: from pathogenesis to therapeutic targets.

As a fundamental part of epitranscriptomics (61, 62), it has gone from simply being seen as an accompanying event to now being regarded as a significant contributing factor in DKD (63, 64). This review shows how m6A regulators become unbalanced in DKD podocytes, specifically through abnormal upregulation of writers METTL3 and METTL14 and the eraser FTO. Current experimental models suggest that these regulatory pathways do not act in isolation; rather, they form an interconnected network. Triggered by high glucose metabolic stress, this systemic epitranscriptomic disruption appears to exert a dual effect: promoting injury pathways while simultaneously impairing cellular defense mechanisms. On one hand, the pathways involving METTL3 and TRIM29, METTL3 and MDM2, as well as FTO and ATF3 drive pyroptosis, apoptosis, and stalled autophagy by stabilizing certain target mRNAs. On the other hand, the pathways linking METTL3 to PINK1 and METTL14 to Sirt1 break down protective mechanisms like mitophagy and antioxidant defense through mRNA degradation. In experimental settings, the release of ROS from accumulated damaged mitochondria and the activation of the NLRP3 inflammasome appear to reinforce each other, creating a pathogenic cycle. This multidimensional epitranscriptomic reprogramming is hypothesized to systematically dismantle podocyte homeostasis, potentially culminating in podocyte detachment and the collapse of the glomerular filtration barrier. The complete pathogenic process is shown in Figure 1. From a translational perspective, the m6A regulatory network holds immense value. On the one hand, m6A regulators like METTL3 and their spectrum of downstream effectors have demonstrated significant potential for the early diagnosis and monitoring of DKD progression. Beyond the hub genes associated with the immune microenvironment such as CTSS and CD53, newly identified targets reflecting tubular injury, metabolic reprogramming, and mitochondrial function, including LRPPRC, DKK3, EGR1, and lncRNA TUG1, collectively construct a multidimensional molecular diagnostic window (27, 50, 52, 54–56). On the other hand, preclinical studies exemplified by the METTL3 inhibitor STM2457 have provided initial confirmation that targeting m6A modifications is an effective therapeutic strategy for alleviating DKD (24, 65). Table 2 shows the novel biomarker targets for podocyte injury in DKD.

**TABLE 2:** m6A epigenetic signatures and emerging biomarker targets in DKD podocyte injury.

New biological targets	Molecular type	m6A Regulatory mechanism (upstream)	Mechanism of action / pathway	Pathological outcome	References
**CTSS**	Coding gene	Closely linked to m6A patterns (esp. immune-infiltrated subtypes); significantly upregulated in DKD.	Bridges of m6A epigenetics and immune microenvironment; mediates immune cell activation and inflammation	**Renal deterioration indicator:** Negatively correlates with eGFR; potential marker for DKD progression and immuno-inflammatory status.	(50)
CD53	Coding gene	Associated with m6A patterns and WTAP expression; significantly upregulated in DKD.	Hub gene regulating B/T cell activation and adhesion within the immune microenvironment.	Renal injury marker: negatively correlates with eGFR; prognostic biomarker for assessing injury severity.	(50)
LRPPRC	m6A Reader / mitochondrial regulator	Downregulated m6A reader; significantly decreased in DKD tubular cells (esp. Loop of Henle).	Maintains mitochondrial mRNA stability, transport, and translation; downregulation causes mitochondrial dysfunction.	Tubular injury alert: low expression predicts progression; correlates positively with eGFR and negatively with proteinuria.	(52)
DDX3Y	Coding gene	Regulated by WTAP and METTL14-mediated m6A; aberrantly expressed in tubular epithelial cells.	Modulates Wnt signaling; participates in tubular stress response and fibrosis.	Tubular stress marker: reflects renal stress under metabolic pressure; allows non-invasive monitoring of pathophysiology.	(30, 53)
EGR1	Coding gene	Regulated by FTO; FTO-mediated demethylation induces aberrant expression.	Activates STING1 signaling; drives the release of inflammatory mediators as a transcription factor.	Inflammatory driver: promotes podocyte injury and renal inflammation; exacerbates DKD progression.	(36)
ACC1	Coding gene	Regulated by FTO; demethylation enhances mRNA stability, causing protein overexpression	Promotes aberrant fatty acid metabolism; leads to intracellular lipid deposition and lipotoxicity	Lipotoxicity marker: reflects lipid dysregulation (esp. in podocytes) driving progression toward end-stage disease.	(57)
lncRNA TUG1	lncRNA	Regulated by METTL3/14; m6A recognized by IGF2BP2 maintains stability. Reduced m6A in DKD causes degradation.	Protective factor regulating PGC-1α for mitochondrial biogenesis/autophagy; degradation leads to loss of protection.	Mitochondrial failure: low expression leads to oxidative stress, senescence, and fibrosis; sensitive indicator of mitochondrial injury.	(54, 55)

DKD, diabetic kidney disease; m6A, N^6^-methyladenosine; CKD, chronic kidney disease; ESRD, end-stage renal disease; CKM, Cardiovascular Kidney Metabolic; eGFR, estimated glomerular filtration rate; GFB, Glomerular Filtration Barrier; GBM, glomerular basement membrane; EMT, epithelial-mesenchymal transition; AGEs, advanced glycation end products; KIM-1, Kidney Injury Molecule 1; NGAL, Neutrophil Gelatinase Associated Lipocalin; TNFR1/2, Tumor Necrosis Factor Receptors 1 and 2.

### 5.1 Challenges in clinical implementation

The translation of m6A biomarkers from bench discovery to routine clinical practice faces several significant barriers. A primary challenge is assay standardization. Current profiling heavily relies on complex sequencing methodologies that are cost prohibitive, technically demanding, and lack the reproducibility required for high throughput clinical laboratories. Developing scalable and standardized diagnostic alternatives is essential. Moreover, there is notable variability and inconsistency between different experimental models, and a critical absence of prospective human validation studies. Biomarker performance must be rigorously tested across diverse diabetic populations to account for confounding variables, including chronic kidney disease staging, systemic inflammation, metabolic heterogeneity, and the effects of concurrent medications (66–68). Future efforts must prioritize large scale multicenter clinical cohort studies to validate the predictive efficacy of these markers in real world patient populations (52, 55).

### 5.2 Unresolved mechanisms and future directions

Beyond clinical validation, the underlying mechanisms of m6A modification are still not fully understood. Target specificity and safety remain major challenges in clinical drug development. Core components like *METTL3* and *FTO* are expressed ubiquitously throughout the body, and their baseline activities are essential for normal physiological processes, including immune response and central nervous system function (69). Consequently, the systemic administration of m6A inhibitors raises significant safety concerns regarding potential off target effects. To achieve therapeutic efficacy while minimizing systemic toxicity, creating kidney specific or podocyte specific targeted delivery systems, such as functionalized lipid nanoparticles is essential. Extensive pharmacokinetic and pharmacodynamic studies are required to establish the therapeutic window and long term safety profile of these interventions before advancing to human clinical trials. Therefore, creating kidney specific or podocyte specific m6A modulators is essential to reduce side effects and improve safety. Key questions also remain regarding how different readers, such as *YTHDFs* versus *IGF2BPs*, compete for the same target (70), and how m6A functions differ across RNA types like mRNA, lncRNA, and circRNA (71). Finally, podocyte injury does not happen in isolation but is central to the DKD microenvironment. Future work should explore how m6A drives communication between podocytes, endothelial cells, and mesangial cells to better understand the full picture of DKD.

## 6 Conclusion

The m6A regulatory network appears to be a crucial component in the pathogenesis of podocyte injury and early cellular failure in DKD. Shifting the focus from pure mechanistic discovery to preclinical translation highlights a promising suite of epitranscriptomic biomarker candidates. If clinically validated, these molecular signatures have the potential to detect disease progression earlier than traditional metrics, such as the estimated glomerular filtration rate. Clarifying this network not only advances our understanding of the molecular underpinnings of the disease but also opens new avenues for non-invasive diagnostics and multi target drug discovery. While significant challenges remain regarding assay standardization and targeted drug delivery, integrating future m6A modulators and diagnostic probes with established clinical regimens holds the promise to bridge the gap between laboratory discovery and clinical practice, ultimately reshaping the management paradigm for this complex disease.
